# Genetic determinants of increased body mass index mediate the effect of smoking on increased risk for type 2 diabetes but not coronary artery disease

**DOI:** 10.1093/hmg/ddaa193

**Published:** 2020-08-24

**Authors:** Christopher S Thom, Zhuoran Ding, Michael G Levin, Scott M Damrauer, Kyung Min Lee, Julie Lynch, Kyong-Mi Chang, Philip S Tsao, Kelly Cho, Peter W F Wilson, Themistocles L Assimes, Yan V Sun, Christopher J O’Donnell, VA Million Veteran Program, Marijana Vujkovic, Benjamin F Voight

**Affiliations:** Division of Neonatology, Children’s Hospital of Philadelphia, Philadelphia, PA 19104, USA; Department of Systems Pharmacology and Translational Therapeutics, Perelman School of Medicine, University of Pennsylvania, Philadelphia, PA 19104, USA; Department of Genetics, Perelman School of Medicine, University of Pennsylvania, Philadelphia, PA 19104, USA; Institute of Translational Medicine and Therapeutics, Perelman School of Medicine, University of Pennsylvania, Philadelphia, PA 19104, USA; Department of Systems Pharmacology and Translational Therapeutics, Perelman School of Medicine, University of Pennsylvania, Philadelphia, PA 19104, USA; Department of Genetics, Perelman School of Medicine, University of Pennsylvania, Philadelphia, PA 19104, USA; Institute of Translational Medicine and Therapeutics, Perelman School of Medicine, University of Pennsylvania, Philadelphia, PA 19104, USA; Department of Biostatistics, Epidemiology and Informatics, Perelman School of Medicine, University of Pennsylvania, Philadelphia, PA 19104, USA; Corporal Michael J. Crescenz VA Medical Center, Philadelphia, PA 19104, USA; Department of Medicine, Perelman School of Medicine, University of Pennsylvania, Philadelphia, PA 19104, USA; Division of Cardiovascular Medicine, Perelman School of Medicine, University of Pennsylvania, Philadelphia, PA 19104, USA; Department of Medicine, Perelman School of Medicine, University of Pennsylvania, Philadelphia, PA 19104, USA; Division of Cardiovascular Medicine, Perelman School of Medicine, University of Pennsylvania, Philadelphia, PA 19104, USA; Department of Surgery, Perelman School of Medicine, University of Pennsylvania, Philadelphia, PA 19104, USA; VA Informatics and Computing Infrastructure, VA Salt Lake City Health Care System, Salt Lake City, UT 84148, USA; VA Informatics and Computing Infrastructure, VA Salt Lake City Health Care System, Salt Lake City, UT 84148, USA; University of Massachusetts College of Nursing & Health Sciences, Boston, MA 02125, USA; Corporal Michael J. Crescenz VA Medical Center, Philadelphia, PA 19104, USA; Department of Medicine, Perelman School of Medicine, University of Pennsylvania, Philadelphia, PA 19104, USA; VA Palo Alto Health Care System, Palo Alto, CA 94304, USA; Department of Medicine, Stanford University School of Medicine, Stanford, CA 94305, USA; VA Boston Healthcare System, Boston, MA 02130, USA; Department of Medicine, Brigham and Women’s Hospital, Boston, MA 02115, USA; Atlanta VA Health Care System, Decatur, GA 30033, USA; Division of Cardiology, Emory University School of Medicine, Atlanta, GA 30322, USA; VA Palo Alto Health Care System, Palo Alto, CA 94304, USA; Department of Medicine, Stanford University School of Medicine, Stanford, CA 94305, USA; Atlanta VA Health Care System, Decatur, GA 30033, USA; Department of Epidemiology, Emory University Rollins School of Public Health, Atlanta, GA 30322, USA; VA Boston Healthcare System, Boston, MA 02130, USA; Department of Medicine, Brigham and Women’s Hospital, Boston, MA 02115, USA; Department of Medicine, Harvard Medical School, Boston, MA 02115, USA; Corporal Michael J. Crescenz VA Medical Center, Philadelphia, PA 19104, USA; Department of Medicine, Perelman School of Medicine, University of Pennsylvania, Philadelphia, PA 19104, USA; Department of Systems Pharmacology and Translational Therapeutics, Perelman School of Medicine, University of Pennsylvania, Philadelphia, PA 19104, USA; Department of Genetics, Perelman School of Medicine, University of Pennsylvania, Philadelphia, PA 19104, USA; Institute of Translational Medicine and Therapeutics, Perelman School of Medicine, University of Pennsylvania, Philadelphia, PA 19104, USA; Corporal Michael J. Crescenz VA Medical Center, Philadelphia, PA 19104, USA

## Abstract

Clinical observations have linked tobacco smoking with increased type 2 diabetes risk. Mendelian randomization analysis has recently suggested smoking may be a causal risk factor for type 2 diabetes. However, this association could be mediated by additional risk factors correlated with smoking behavior, which have not been investigated. We hypothesized that body mass index (BMI) could help to explain the association between smoking and diabetes risk. First, we confirmed that genetic determinants of smoking initiation increased risk for type 2 diabetes (OR 1.21, 95% CI: 1.15–1.27, *P* = 1 × 10^−12^) and coronary artery disease (CAD; OR 1.21, 95% CI: 1.16–1.26, *P* = 2 × 10^−20^). Additionally, 2-fold increased smoking risk was positively associated with increased BMI (~0.8 kg/m^2^, 95% CI: 0.54–0.98 kg/m^2^, *P* = 1.8 × 10^−11^). Multivariable Mendelian randomization analyses showed that BMI accounted for nearly all the risk smoking exerted on type 2 diabetes (OR 1.06, 95% CI: 1.01–1.11, *P* = 0.03). In contrast, the independent effect of smoking on increased CAD risk persisted (OR 1.12, 95% CI: 1.08–1.17, *P* = 3 × 10^−8^). Causal mediation analyses agreed with these estimates. Furthermore, analysis using individual-level data from the Million Veteran Program independently replicated the association of smoking behavior with CAD (OR 1.24, 95% CI: 1.12–1.37, *P* = 2 × 10^−5^), but not type 2 diabetes (OR 0.98, 95% CI: 0.89–1.08, *P* = 0.69), after controlling for BMI. Our findings support a model whereby genetic determinants of smoking increase type 2 diabetes risk indirectly through their relationship with obesity. Smokers should be advised to stop smoking to limit type 2 diabetes and CAD risk. Therapeutic efforts should consider pathophysiology relating smoking and obesity.

## Introduction

Obesity and type 2 diabetes are leading causes of death worldwide (1). These conditions and related comorbidities are global epidemics, expected to place ever larger demands on health care systems. As such, revealing the underlying causal risk factors and their associated biological pathways are crucial for public health.

Epidemiologic (2–6) studies have associated smoking with increased diabetes risk. Indeed, smoking can perturb glycemic regulation (5,7). However, smoking also impacts inflammatory processes (8,9) and a number of cardiometabolic traits, including obesity, blood pressure and heart disease (10–12). Some studies have shown that body mass index (BMI) increases smoking risk (10), whereas others have suggested that smoking may actually decrease BMI (11,13,14). An improved understanding of the mechanisms underlying the link between smoking, obesity and diabetes may inform targeted therapeutic development and clinical decision-making.

Recent collections of genetic data for smoking behavior, type 2 diabetes and cardiometabolic factors, such as BMI, provide vehicles through which evidence of causality can be evaluated using the framework of Mendelian randomization (MR). Formally, MR uses genetic variants associated with an exposure of interest (i.e. smoking behavior) to create an instrumental variable to estimate a potential causal effect of genetic variants on exposures and outcomes (i.e. type 2 diabetes). Because alleles are randomly allocated at meiosis, and due to the fact that genotype precedes phenotype, this approach with care can address issues of confounding and reverse causality that limit inference in prospective or cross-sectional cohort studies (15). Multivariable MR and causal mediation analyses extend the method to identify and account for additional causal factors that may help explain associations observed in univariable experimental analyses (16,17).

Here, we used MR analysis on summary statistics, as well as individual-level data from the Million Veteran Program (MVP) focused on individuals of European ancestry, to define relationships between smoking and cardiometabolic traits. We hypothesized that the relationship between smoking and type 2 diabetes was mediated by BMI. In and in this study, we aimed to (i) estimate the effect of genetically predicted smoking traits on type 2 diabetes, (ii) compare the effect of smoking behavior on type 2 diabetes with the effect of smoking on coronary artery disease (CAD), (iii) estimate the effect of smoking on BMI and (iv) determine if BMI mediates the effects of smoking on type 2 diabetes and/or CAD. Our findings elucidate causal genetic effects between these clinically important traits. Better understanding these biological associations will inform clinical management and aid translational research efforts.

## Results

### Increased genetically determined odds of smoking initiation elevates type 2 diabetes risk

**Figure 1:**
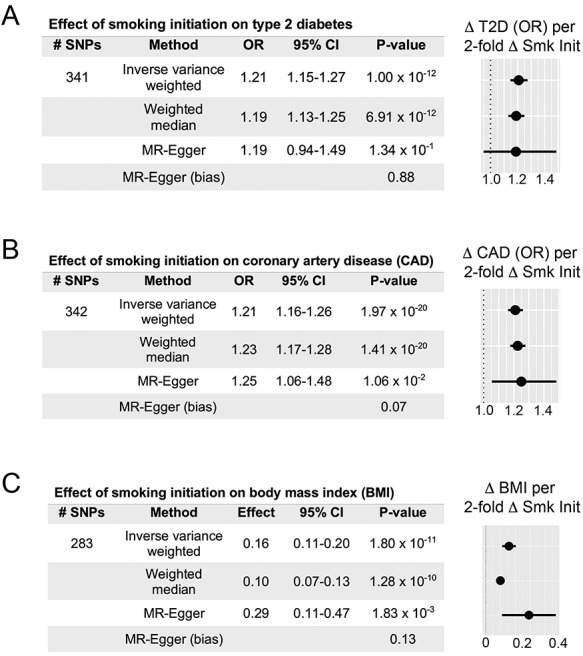
Two-sample Mendelian randomization determines that smoking initiation (Smk Init) increases type 2 diabetes (T2D) risk, CAD risk and BMI. (**A**) Genetically determined smoking initiation risk increases T2D risk. Two-sample Mendelian randomization OR estimates, 95% confidence intervals and forest plot represent changes associated with 2-fold increase in genetic smoking initiation ‘risk’. MR-Egger intercept, a bias measurement, does not deviate significantly from zero. This validates effect estimates. (**B**) Increased smoking initiation risk elevates CAD risk. OR estimates, 95% confidence intervals and forest plot represent changes associated with 2-fold increase in smoking initiation exposure. MR-Egger intercept does not deviate significantly from zero, validating the effect estimate. (**C**) Increased smoking initiation risk increases BMI. Effect estimates, 95% confidence intervals and forest plot represent changes in BMI (standard deviation units) associated with 2-fold increase in smoking initiation exposure. MR-Egger intercept does not deviate significantly from zero, validating the effect estimate.

First, we assessed if genetically determined smoking initiation, smoking cessation and smoking frequency (number of cigarettes per day) modulated susceptibility to type 2 diabetes. To estimate a causal effect between odds of smoking initiation and susceptibility to type 2 diabetes, we performed causal inference analysis using MR. Using summary statistics obtained from genome-wide association studies (GWAS) of these traits, we generated an instrumental variable (IV) for smoking initiation (18) and tested its association with type 2 diabetes (19). Our genetic instrument comprised 341 linkage-independent single nucleotide polymorphisms (SNPs, EUR *r*^2^ < 0.01) that met genome-wide significance for smoking initiation. This instrumental variable was not subject to weak instrument bias, nor were others used in this study (Supplementary Material, Table S1) (20). We observed that both inverse variance weighted (*P* = 1.0 × 10^−12^) and median weighted (*P* = 6.9 × 10^−12^) MR methods demonstrated statistically significant positive association between increased genetically determined odds of smoking initiation and susceptibility to type 2 diabetes (Fig. 1A). As a sensitivity analysis, we performed the MR-Egger regression test and found no evidence of systematic bias in our estimated effect (MR-Egger intercept term *P* = 0.88). These data support the hypothesis that increased odds of smoking initiation corresponds with an increase in odds of type 2 diabetes risk; each 2-fold increase in genetic predisposition to smoking initiation corresponding to a 21% increased odds of type 2 diabetes risk (OR 1.21, 95% confidence interval = 1.15–1.27, by inverse variance weighted method). This effect size was consistent with a recent study (21).

### Smoking cessation and frequency were not associated with type 2 diabetes risk

We next generated instrumental variables for additional smoking behaviors, including smoking cessation and smoking frequency (cigarettes per day). The number of SNPs comprising the instruments for these traits were substantially smaller (*n* = 18 and *n* = 42 SNPs, respectively). We observed no evidence of association with either of these traits and type 2 diabetes risk (Supplementary Material, Fig. S1 and Supplementary Material, Table S2). A lack of association for these smoking traits with type 2 diabetes risk may be driven by lack of statistical power to detect relatively small effect sizes. Indeed, an instrumental variable based on lifetime smoking exposure (22), which accounts for smoking frequency and duration, identified significant effects on type 2 diabetes (Supplementary Material, Table S3).

### Smoking traits are genetically correlated with type 2 diabetes risk

If the results from the above were merely due to a lack of statistical power and not a null association, we still might expect to observe positive genetic correlation between smoking behaviors and type 2 diabetes risk. Therefore, we quantified the extent to which genetic susceptibility to these smoking traits correlated with genetic susceptibility to type 2 diabetes. Using linkage disequilibrium score regression (LDSC) (23), we observed significant and positive genetic correlations between smoking traits and type 2 diabetes (Supplementary Material, Table S4). These results suggest that future genetic studies that explain more of the genetic variance in smoking behavior and type 2 diabetes might clarify the effects of these smoking traits on type 2 diabetes.

### Smoking initiation may increase HbA1c levels

As a sensitivity analysis, we estimated the causal effect of smoking initiation on genetically determined HbA1c levels (24). HbA1c level represents glycated hemoglobin and is used to clinically diagnose type 2 diabetes. As such, HbA1c level is a biochemical surrogate for type 2 diabetes. We created a genetic instrument comprised of 282 SNPs, and observed that a 2-fold increase in genetically determined smoking risk was associated with a 0.02 standard deviation unit greater HbA1c level (95% CI: 0.01–0.03, *P* = 3.1 × 10^−3^ by inverse variance weighted method, Supplementary Material, Fig. S2). Although this association was not significant in weighted median or MR-Egger tests, these findings gave additional support for a relationship between smoking behavior and glycemic regulation (5,7) and between genetically determined smoking risk and type 2 diabetes.

### Increased risk of type 2 diabetes by smoking initiation is equivalent to that of CAD

To contrast our estimated effect with established causal relationships, we next used MR to address the effect of smoking initiation on susceptibility to CAD. We observed a positive causal effect from smoking on CAD risk (Fig. 1B and Supplementary Material, Table S3), with a 2-fold increase in smoking initiation risk increasing odds of CAD risk by 21% (95% CI: 1.16–1.26, *P* = 2.0 × 10^−20^). This estimated causal effect on CAD was consistent with previous reports (6). We observed that the estimated effect of smoking initiation on CAD risk was quite similar to increased type 2 diabetes risk from this behavior (Fig. 1A). These results suggested that smoking initiation portends an equivalently increased risk of type 2 diabetes and CAD. However, it remained to be seen whether biological factors shared between diseases mediate the observed associations.

### Genetically elevated smoking initiation risk is associated with increased BMI

Prior evidence has demonstrated a positive correlation between smoking and BMI (10,12,25), although some genetic results have indicated that BMI might actually decrease as a result of smoking (11,14). This latter finding is consistent with reported clinical observations that linked smoking cessation with weight gain (26). We hypothesized that BMI mediates the effects of smoking on type 2 diabetes and/or CAD risk.

We first tested the causal association of BMI with smoking initiation and demonstrated that a 1364-SNP instrumental variable based on BMI (27) increased smoking initiation risk, with a 25% increased odds of smoking initiation per standard deviation unit increase in genetically determined BMI (95% CI: 1.21–1.29, *P* = 2.1 × 10^−44^, Supplementary Material, Fig. S3). We noted that the MR-Egger intercept significantly deviated from zero, suggesting the presence of negative, directional horizontal pleiotropy (*P* = 1.1 × 10^−4^, Supplementary Material, Fig. S3). Results using methods more robust to the presence of pleiotropy (e.g. weighted median and MR-Egger) demonstrated significant effects of BMI on smoking, though effect estimates differed (Supplementary Material, Fig. S3). Overall, these results confirmed the previously reported genetic association (10,12).

Next, we conversely assessed whether genetic risk of initiating smoking behavior was associated with BMI. Indeed, there was a positive association between smoking and BMI (Fig. 1C and Supplementary Material, Table S3), with each 2-fold increase in genetically determined smoking initiation risk corresponding to a 0.16 standard deviation unit (~0.8 kg/m^2^) increase in BMI (95% CI: 0.11–0.20, *P* = 1.8 × 10^−11^). We noted that the MR-Egger intercept was not significantly different from zero (*P* = 0.13), indicating little if any evidence of directional bias on this estimated effect (Fig. 1C). Our findings indicated a significant, shared genetic risk between smoking and BMI. From a biological standpoint, these results supported prior studies relating nicotine craving to a desire to overeat (12).

### BMI mediates the effect of smoking initiation risk on type 2 diabetes

**Figure 2:**
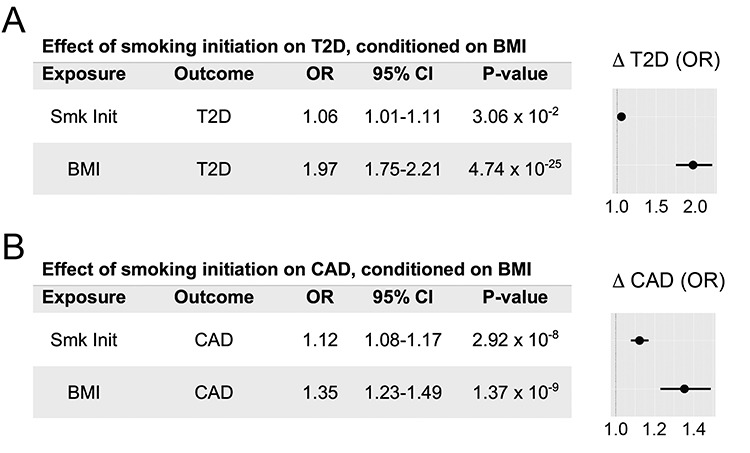
BMI largely mediates the effect of smoking initiation on increased type 2 diabetes (T2D) risk, but not CAD risk. Instrumental variables for these experiments comprised ~286 SNPs from smoking initiation GWAS summary statistics (18). (**A**) Multivariable Mendelian randomization results show that genetically determined BMI largely confounds the effect of smoking initiation on T2D risk. (**B**) Multivariable MVMR results show that smoking initiation retains a strong independent effect on CAD risk after conditioning on BMI. OR estimates, 95% confidence intervals and forest plots represent changes in outcomes associated with 2-fold increase in genetic smoking initiation ‘risk’, conditioned on BMI.

We reasoned that smoking effects on BMI could mediate the potentially complex relationship between smoking and type 2 diabetes. We therefore estimated causal effects of smoking on type 2 diabetes risk, accounting for BMI, using multivariable MR. Our results showed that the effects of smoking on type 2 diabetes are largely explained by BMI. When including an effect from BMI in the model, the residual independent (direct) effect of smoking on type 2 diabetes was attenuated (OR 1.06, 95% CI: 1.01–1.11, *P* = 0.03, Fig. 2A). In an analogous experiment, we found that the effect of smoking initiation on HbA1c level was no longer significant (*P* = 0.24, Supplementary Material, Fig. S4). Furthermore, an instrument based on lifetime smoking exposure, accounting for smoking frequency and duration (22), showed qualitatively similar results (OR 1.25, CI: 0.97–1.62, *P* = 0.09, Supplementary Material, Fig. S5A). These results demonstrated that BMI may account for most of the effect of genetically determined risk of smoking initiation on increased type 2 diabetes risk.

We next considered the reciprocal experiment, using an instrumental variable (>1300 SNPs) based on BMI, to estimate effects of BMI while accounting for smoking behavior on type 2 diabetes susceptibility or HbA1c levels (Supplementary Material, Fig. S6A and B). BMI is an established, causal risk factor for type 2 diabetes (28), and the traits share a common genetic basis (Supplementary Material, Table S4). As expected, we found that a one standard deviation increase in genetically determined BMI corresponded to a 2.5-fold increase in diabetes risk (95% CI: 2.36–2.70, *P* = 6.6 × 10^−134^). However, smoking was not independently associated with type 2 diabetes (*P* = 0.54). Similarly, a one standard deviation unit increase in genetically determined BMI was associated with a 0.06 unit increase in HbA1c (95% CI: 0.05–0.07, *P* = 4.2 × 10^−24^) but smoking initiation was not associated with HbA1c levels (*P* = 0.14). Multivariable MR experiments using a combined instrument with 1410 SNPs significant for smoking or BMI, which was heavily weighted toward BMI-associated SNPs, revealed similar findings (Supplementary Material, Table S5). An attenuated yet significant association between lifetime smoking and type 2 diabetes remained after accounting for BMI. These results demonstrated that BMI contributes substantial effects on increased type 2 diabetes risk, separately from smoking behavior.

Sample overlap across GWAS can bias MR results (29), and all GWAS used to generate our results up to this point had included samples from the UK Biobank. We, therefore, performed additional analyses of smoking initiation (18) or lifetime smoking score (22) using studies for BMI (30) and type 2 diabetes risk (31) that analyzed nonoverlapping sample populations. Despite reduced sample sizes and power in these experiments (Supplementary Material, Table S1), our findings supported a causal effect of smoking behavior on type 2 diabetes, as well as bidirectional positive effects between smoking behavior and BMI (Supplementary Material, Table S6). There was evidence of significant horizontal pleiotropy in only one instance (lifetime smoking score on type 2 diabetes). Multivariable MR experiments using nonoverlapping data also supported BMI as an important mediator between smoking behavior and type 2 diabetes (Supplementary Material, Table S7). In sum, these sensitivity analyses using nonoverlapping data confirmed that BMI mediates the effect of smoking on type 2 diabetes risk.

### BMI does not fully mediate the effect of smoking initiation risk on CAD

We then asked whether the effects of smoking on CAD were similarly mediated by BMI. As with BMI and type 2 diabetes, we observed significant genetic correlation between BMI and CAD (*r*_g_ = 0.23, *P* = 3.0 × 10^−24^, Supplementary Material, Table S4). In contrast to type 2 diabetes, there remained a significant independent effect of smoking initiation on CAD risk after controlling for BMI (OR 1.12, 95% CI: 1.08–1.17, *P* = 2.9 × 10^−8^, Fig. 2B). Analyses using genetic instruments based on lifetime smoking exposure (OR 1.72, CI: 1.43–2.07, *P* = 9.2 × 10^−8^, Supplementary Material, Fig. S5B), BMI (OR 1.23, CI: 1.14–1.32, *P* = 7.9 × 10^−8^, Supplementary Material, Fig. S6C), or combined BMI- and smoking initiation-associated SNPs (OR 1.20, 95% CI: 1.14–1.26, *P* = 7.0 × 10^−13^, Supplementary Material, Table S8) all showed a persistent effect of smoking on susceptibility to CAD. This suggests that the biology underlying the association between smoking and CAD partially involves obesity-related pathways, but smoking behavior is still associated with direct effects on atherosclerosis through contributions from additional factors.

As a sensitivity analysis, we also confirmed these results in experiments for smoking initiation (18) or lifetime smoking score (22) using studies of BMI (30) and CAD (32) that analyzed nonoverlapping sample populations. These experiments confirmed the significant association between smoking behavior and CAD without evidence of horizontal pleiotropy (Supplementary Material, Table S6), as well as an independent effect of smoking on CAD that persisted after correcting for effects of BMI (Supplementary Material, Table S9).

### Smoking increases type 2 diabetes risk via BMI and independently increases CAD risk

**Figure 3:**
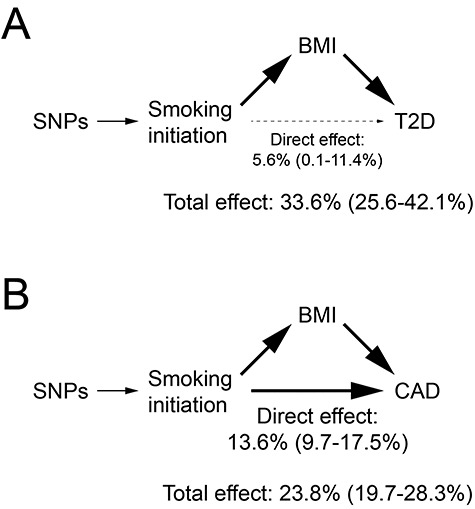
Models for how genetically determined smoking initiation risk impacts cardiovascular disease traits. (**A**) SNPs determine genetic risk of smoking initiation. By MR-Steiger estimates (33), smoking initiation directionally influences BMI. From mediation analysis (17), the total and direct effects of smoking initiation on type 2 diabetes risk (T2D) are shown, representing increased odds of T2D (with 95% confidence interval) per 2-fold increase in genetically determined smoking initiation risk. (**B**) Both smoking initiation and BMI have strong independent effects on CAD risk. Total and direct effects of smoking initiation on CAD are shown, representing increased odds of CAD (with 95% confidence interval) per 2-fold increase in genetically determined smoking initiation risk.

We sought to assess the directionality of the estimated causal effect between smoking, BMI and type 2 diabetes. MR-Steiger can infer the direction of causality using GWAS summary statistics in situations where the biology of underlying SNPs is not yet understood (33). We used MR-Steiger to test whether the multi-trait relationships seen in our analyses were best explained by smoking initiation or BMI as driving associations with type 2 diabetes. For this experiment, we analyzed 1085 SNPs that met genome-wide significance (*P* < 5 × 10^−8^) in GWAS for smoking initiation and BMI (27). We determined that our findings were best explained by a model in which smoking increased BMI (MR-Steiger sensitivity 14.95, *P* < 10^−10^, Fig. 3).

Next, we used mediation analyses to estimate the total and direct effects of smoking on type 2 diabetes and CAD risks (17). In mediation analysis, binary outcomes preclude accurate estimation of indirect effects (17). We found that the total effect of a 2-fold genetically increased smoking initiation risk was to increase the odds of type 2 diabetes risk by 33.6% (95% CI: 25.6–42.1%), although the direct effect attributable to smoking initiation was just 5.6% (95% CI: 0.1–11.4%, Fig. 3A). In contrast, the total effect of increased smoking initiation was to increase CAD risk by 23.8% (CI: 19.7–28.3%), with a direct effect from smoking initiation of 13.6% (95% CI: 9.7–17.5%, Fig. 3B). Mediation analyses based on lifetime smoking score, and/or less well-powered, nonoverlapping data sets, showed consistent effect patterns (Supplementary Material, Tables S10), with equivocal directional causality between smoking initiation and BMI (Supplementary Material, Table S11). In sum, these results demonstrated that the majority of the effect from smoking on type 2 diabetes was mediated by BMI, whereas both smoking behavior and BMI independently affect CAD risk.

### Individual-level MR studies confirm that BMI mediates the effects of smoking on type 2 diabetes, but not CAD

Finally, to replicate the relationships between smoking behavior and cardiovascular traits observed in our two-sample MR experiments described above, we analyzed individual-level data in 225 252 individuals of European ancestry from the MVP (34,35). To create a genetic instrument that predicted smoking behavior, we assembled a set of linkage independent (*r*^2^ < 0.01), genome-wide significant SNPs (*P* < 5 × 10^−8^) associated with smoking initiation (*n* = 290) or lifetime smoking (*n* = 205). When applied to the MVP population, this polygenic risk score (PRS) explained 0.3% of the variance in smoking initiation, as defined as ‘ever’ or ‘never’ having smoked (*P* < 2 × 10^−16^). We determined this instrument was not subject to weak instrument bias (*F*-statistic = 380).

**Figure 4:**
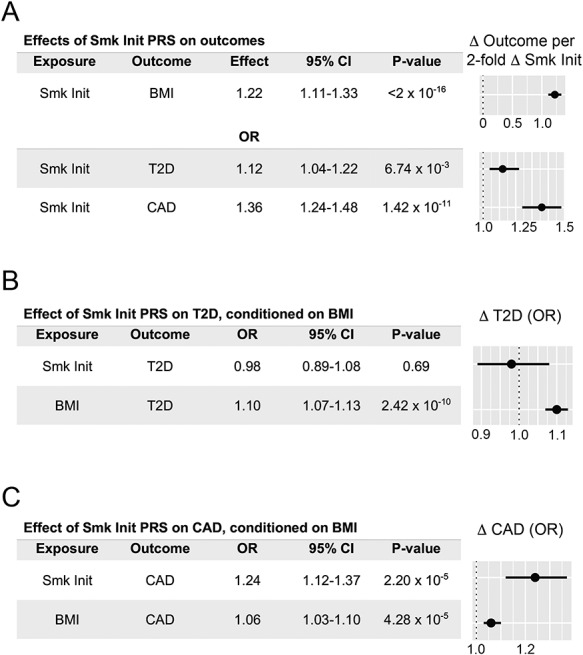
Individual-level Mendelian randomization analyses confirm that a polygenic risk score for smoking initiation increases cardiovascular risks, and that BMI mediates the effect of smoking on type 2 diabetes but not CAD. (**A**) A PRS based on genome-wide significant SNPs for smoking initiation increases BMI, type 2 diabetes (T2D) or CAD risks. (**B**) Effects from smoking initiation on type 2 diabetes are nonsignificant after adjusting for effects on BMI. (**C**) Effects from smoking initiation on CAD remain significant after adjusting for BMI. OR and effect estimates, 95% confidence intervals and forest plots represent changes in outcomes associated with 2-fold increase in genetic smoking initiation ‘risk’.

Next, we confirmed the association with our genetic instrument for predicted smoking behavior on BMI, type 2 diabetes, or CAD, using a two-stage predictor substitution procedure. In the first-stage analyses, after adjusting for age, sex and previously validated ancestry-related principal components (35,36), we found that a 2-fold increase in genetically determined smoking initiation risk was associated with increases in BMI (effect: 1.22 kg/m^2^, 95% CI: 1.11–1.33, *P* < 2 × 10^−16^), type 2 diabetes risk (OR 1.12, 95% CI: 1.04–1.22, *P* = 6.7 × 10^−3^) and CAD (OR 1.36, 95% CI: 1.24–1.48, *P* = 1.4 × 10^−11^, Fig. 4A). Experiments using a lifetime smoking score-related PRS, and/or BMI effects weighted based on findings from an older BMI GWAS (30), returned qualitatively analogous results (Supplementary Material, Table S12).

Then, we included the effect of our genetic instrument of smoking behavior on BMI to estimate the remaining effect on both type 2 diabetes and CAD. In the second-stage analyses that adjusted for BMI, there remained no significant effect of smoking initiation on type 2 diabetes in this population (*P* = 0.69, Fig. 4B and Supplementary Material, Table S13). Conversely, there remained strong independent effects for smoking initiation on CAD after accounting for BMI (OR 1.24, 95% CI: 1.12–1.37, *P* = 2.2 × 10^−5^, Fig. 4C and Supplementary Material, Table S14). Experiments based on a lifetime smoking score-related PRS and/or BMI weights associated with an older BMI GWAS (30) confirmed these trait relationships (Supplementary Material, Tables S13 and S14). As there is no overlap between exposure and outcome sample populations for these individual level experiments, these results represent a direct replication of observations made in the two-sample MR experiments described above. In sum, these findings strongly support the inference that BMI mediates the effect of smoking behavior on type 2 diabetes, but not the effects of smoking on heart disease.

## Discussion

Despite long-standing clinical observations (6), a causal genetic association between smoking and type 2 diabetes has only recently been established (21). Our results indicate a more complex relationship between smoking and type 2 diabetes than what has been reported previously (1,2,21,37). We associated a 2-fold increased smoking initiation risk with a ~1.2 kg/m^2^ greater BMI (in MVP). Genetically determined smoking initiation risk increases type 2 diabetes risk and HbA1c, but these associations are mostly if not entirely mediated through an association between smoking on BMI. Our findings were consistent across instrumental variable methodological approaches, and we did not observe evidence of systematic biases to our effect estimate. By clarifying the biological factors underlying these trait associations, our findings will allow for more informed clinical recommendations and improve targeted therapeutic development.

Our findings support a bidirectional positive causal relationship between smoking behavior and BMI. In fact, some results suggested that smoking behavior may drive increased BMI, as opposed to BMI driving an increased desire to smoke (Fig. 3). However, the relationship between these traits is biologically complex, and there may be additional effects outside of smoking initiation that influence type 2 diabetes independent of the association with BMI. Future work is needed to clarify the interrelated influences of these traits.

MR analyses can be biased if related data come from GWAS with shared samples (29). Although this risk is reduced in very large studies from international consortia (29), we wanted to control for this potential bias in our study. Indeed, the most recent GWAS for traits considered in our study all included UK Biobank samples (18,19,27,38). For this reason, in sensitivity analyses, we analyzed genetic data for exposures from GWAS with nonoverlapping sample populations. In addition, we performed individual-level experiments using data from the MVP. Those samples were not included in BMI or smoking behavior discovery data sets nor did they contribute to the association studies we used for type 2 diabetes and CAD in the two-sample MR experiments. Thus, our results represent direct replication of those findings. Importantly, all of our results supported our conclusions that (i) there exists a positive bidirectional relationship between smoking and BMI, (ii) BMI mediates much of the effect of smoking on type 2 diabetes risk and (iii) smoking has a strong independent effect on heart disease risk.

Our findings add to numerous reports regarding the negative effects of smoking on cardiometabolic health identified by medical practitioners and policymakers. At minimum, our findings indicate that increased genetic risk of smoking initiation is a key behavior that portends adverse cardiovascular outcomes. Future work is needed to determine how much other specific smoking behaviors, such smoking frequency or rebound effects of cessation (14), underlie cardiovascular morbidities. A recent report detailed one mechanism directly linking smoking with increased blood glucose and type 2 diabetes risk (39). We anticipate that important biological pathways for this association will involve altered obesity and/or adiposity. Indeed, our findings indicate that future validation research should focus on such pathways to establish relevant biological mechanisms.

The association between smoking and type 2 diabetes was similar in magnitude to the association between smoking and CAD. Interestingly, the effect of smoking on CAD risk was only partially mediated by BMI. Although outside the scope of our current study, it will be important for future work to identify how genetically determined smoking risk portends increased CAD risk. Indeed, smoking has myriad deleterious effects, including but not limited to blood pressure elevation, endothelial damage and enhanced inflammation (40). Direct effects from smoking on atherosclerosis may underlie this strong genetic association. Further revealing alternative pathways and risk factors that account for these findings may have translational benefit for diagnosing and treating heart disease in smokers.

In sum, our findings reinforce the importance of clinical recommendations to avoid or stop tobacco smoking. By elucidating genetic factors mediating the associations between smoking behavior, BMI, type 2 diabetes and CAD, our results will inform strategies to mitigate these risks though development of novel therapeutics. Although interpretation of these findings is limited to individuals of European ancestry, we expect that well-powered multi-ethnic GWAS will ultimately confirm this result in other populations.

## Materials and Methods

### Genome-wide summary statistics collection

We analyzed publicly available GWAS summary statistics for smoking initiation (*n* = 1 232 091 individuals), smoking cessation (*n* = 547 219), cigarettes per day (*n* = 337 334) (18), type 2 diabetes (*n* = 898 130) (19), HbA1c (*n* = 123 665) (24), body mass index (BMI, *n* = ~700 000) (27) and coronary artery disease (CAD*, n* = 547 261) (38). Lifetime smoking index was based on UK Biobank data (*n* = 462 690) (22). To eliminate potential bias from sample overlap across GWAS (29), we also performed analyses using findings from nonoverlapping sample populations for type 2 diabetes (*n* = 291 748) (31), CAD (*n* = 184 305) (32) and BMI (*n* = 339 224) (30). All data sets were from individuals of European ancestry only and were analyzed in genome build hg19/GRCh37.

Smoking initiation was defined as having smoked ‘regularly’, every day for at least 1 month or > 100 cigarettes in one’s lifetime (18). Smoking cessation was defined as those who were identified as having initiated smoking but subsequently stopped. Smoking frequency (cigarettes per day) was defined as the number of cigarettes smoked per day, on average, as a current or former smoker. Lifetime smoking index scores, which take into account smoking initiation, frequency and duration, were previously calculated (22).

### Genetic variant selection related to smoking exposures

We created genetic instrumental variables (IVs) for various smoking traits, including ‘smoking initiation’, ‘smoking cessation’, ‘cigarettes per day’ (smoking frequency) (18) and lifetime smoking exposure (22), as well as HbA1c (24) and BMI (27). To generate IVs, we first identified SNPs common to both exposure and outcome data sets. Using two-sample MR, we then clumped all genome-wide significant SNPs to identify those within independent linkage disequilibrium blocks (EUR *r*^2^ < 0.01) in 250 kb regions. Full data sets for our IVs are shown in Supplementary Material, Tables S15–S48.

We used mRnd (http://cnsgenomics.com/shiny/mRnd/) (20) to estimate the strength (*F*-statistics) of these IVs. Smoking initiation or cessation were input as binary exposure variables. All other traits were analyzed as continuous exposure variables. We calculated proportion of genetic inheritance explained in each exposure per Shim *et al.* (41). None of our instrumental variables was subject to weak instrument bias, as each had an *F*-statistic greater than 10 (Supplementary Material, Table S1).

### Mendelian randomization and causal effect estimation

We performed two-sample MR (TwoSample MR package v0.5.3) (15) using R (v3.6.1). We present causal estimates from inverse variance weighted (random effects model), weighted median and MR-Egger regression methods. We analyzed for pleiotropic bias using MR-Egger regression intercepts, wherein significant nonzero intercepts can imply directional bias among IVs (42). We performed multivariable Mendelian randomization (MVMR) analyses using the MVMR package (16) in R, and present causal estimates for each associated variable. For causal direction analysis, we used MR-Steiger and report values for sensitivity, statistical significance and ‘correct causal direction’ (33).

### Genetic correlation estimates

Genetic correlations were estimated using Linkage Disequilibrium Score Regression (LDSC) (23). Summary statistics were munged and analyzed for each trait. Presented data reflect genetic correlation estimates (i.e. *r*_g_ values) and related statistical significance estimates (*P*-values).

### Statistical analysis

In MR analyses, estimated effects from exposure(s) on outcome are presented from inverse variance weighted, weighted median, and MR-Egger regression measures. Because Cochran’s Q test (included in the TwoSample MR package v0.5.3) (15) found heterogeneity in some IVs, we utilized the random-effect model when performing inverse variance weighted MR. We also performed a sensitivity analysis using IV pruning via MR-PRESSO (43) for a single trait pair (smoking initiation and type 2 diabetes). The resultant IV included 325 SNPs from an initial 341, but MR results were not qualitatively different than the full instrument (data not shown). Thus, we performed and report MR results using IVs that had not undergone pruning. Statistical significance was defined as *P* < 0.05 for all experiments.

For continuous outcomes (BMI and HbA1c), results are presented as beta effect values representing changes in standard deviation units for these traits. For the dichotomous outcomes smoking initiation, type 2 diabetes, and CAD, we converted causal effect estimates into odds ratios using previously described methods (44). By multiplying causal effect estimates by ln(2) and taking the exponentiated value (=exp^[ln(2)*effect]), we calculated the change in outcome odds ratio that corresponded with a 2-fold change in genetically determined dichotomous exposure risk. For continuous exposure variables, we exponentiated the causal effect estimate (=exp^[effect]) to reach a value reflecting the change in outcome per standard deviation unit increase in exposure.

### Mediation analysis

Mediation analysis estimates were calculated as described by Burgess *et al* (17). The outcome variables analyzed (type 2 diabetes and CAD risk) are defined in noncollapsible odds ratios. We therefore present only total and direct effects. Indirect effects are inaccurate since there cannot be linear relationships between exposures, mediators and these binary outcomes (17).

### Individual-level statistical data collection and analyses

We used individual-level statistics from the MVP, a large cohort of fully consented veterans from Department of Veteran Affairs facilities (34). Genotyping, quality control and phenotyping for type 2 diabetes and CAD were as previously described (35). Smoking status was defined based on self-reported values of ‘ever’ or ‘never’ having smoked. The top 30 principal components (PCs) were computed using FlashPCA (45) in all MVP participants and an additional 2504 individuals from 1000 Genomes. Self-reported race and genetically inferred ancestry were then harmonized into ancestral groups using a unifying classification algorithm named HARE (36). Principal components were then regenerated in European subjects only, and the first five PCs were used as covariates in all analyses.

To generate our genetic instrument risk scores, we identified SNPs with genome-wide significant effects on smoking initiation (*n* = 3484) or lifetime smoking score (*n* = 4466) that were imputed in MVP data. After clumping (*r*^2^ < 0.01 in 250 kb regions), there remained 290 independent SNPs associated with smoking initiation and 205 independent SNPs associated with lifetime smoking score. We validated that these instruments predicted smoking status in the MVP cohort using a logistic regression model and thus defined the variability in smoking explained via Nagelkerke’s *r*^2^.

We then performed a two-stage predictor substitution (2SPS) (46) analysis using each genetic instrument risk score to estimate the effects of smoking behavior on BMI, type 2 diabetes or CAD. In stage 1, we fit a logistic regression model adjusted for age, sex and European-specific principal components to estimate the effect of the genetic instrument for smoking behavior on smoking status, taking the predicted effect of genetically proxied ‘smoking on smoking’ into the next stage. In stage 2, the causal effect of predicted smoking on type 2 diabetes and CAD was estimated using a logistic regression model. In this model, the predicted smoking status by smoking PRS effect estimate represented the total effect of smoking on each outcome.

We then repeated the 2SPS analysis, this time accounting for both predicted smoking and predicted change in BMI as a consequence of genetic smoking risk, to decompose the direct and indirect effect of smoking on type 2 diabetes and CAD. Stage 1 of this analysis consisted of an additional linear regression model, where the outcome was standardized BMI. The independent genetic predictor in this analysis consisted of the same SNPs used for the smoking genetic risk score (e.g. *n* = 205 and 290 for smoking initiation and lifetime smoking score, respectively). However, the respective SNP weights were derived from two BMI GWAS studies [either Yengo *et al*. (27) or Locke *et al*. (30)]. This genetic risk score ‘BMI-by-smoking-SNPs’ represented the genetically predicted change in BMI caused by cumulative carriership of smoking risk alleles. BMI-by-smoking-SNPs were then used as the instrument to predict BMI, using linear regression including the covariates of age, gender and five PCs. In stage 2, we fit logistic regression models, with type 2 diabetes or CAD as dependent variables, with both ‘predicted smoking by smoking genetic risk score’ and ‘predicted BMI as a consequence of smoking SNPs’ as covariates, along with age, sex and five PCs. In this analysis, the predicted smoking by smoking genetic risk score represented the direct effect of smoking on outcome, whereas predicted BMI as a consequence of smoking SNPs represented the indirect effect of smoking on outcome.

As with summary statistics, the estimated effect sizes were converted to odds ratios of type 2 diabetes or CAD per 2-fold increase in smoking initiation risk (a binary exposure), or odds of types 2 diabetes or CAD per 1 standard deviation increase in lifetime smoking score (a continuous variable). Absolute changes in BMI (in units of kg/m^2^) per 2-fold increased smoking initiation risk or one standard deviation unit increase in lifetime smoking score were calculated by multiplying effect sizes by ln (2).

### Coding scripts and data sets

All relevant coding scripts and data sets can be found on Github (https://github.com/thomchr/SmkT2D). All data and coding scripts are also available upon request.

## Supplementary Material

SmkBMIT2D_HMG_200819_Supp_ddaa193

SmkBMIT2D_HMG_200710_SupplementalTables_ddaa193
