# Supplementary material for: Genetic determinants of increased body mass index mediate the effect of smoking on increased risk for type 2 diabetes but not coronary artery disease
Source: Hum Mol Genet. 2020 Aug 24;29(19):3327–37. doi: 10.1093/hmg/ddaa193 (PMC7689293; doi:10.1093/hmg/ddaa193)
Supplement: SmkBMIT2D_HMG_200819_Supp_ddaa193 [file smkbmit2d_hmg_200819_supp_ddaa193.docx]

**Genetic determinants of increased body mass index mediate the effect of smoking on increased risk for type 2 diabetes risk but not coronary artery disease**

Christopher S Thom ^1,2,3,4,+^, Zhuoran Ding ^2,3,4,5,+^, Michael G Levin ^6,7,8^,

Scott M Damrauer ^7,8,9^, Kyung Min Lee^10^, Julie Lynch^10,11^, Kyong-Mi Chang ^6,7^

Philip S Tsao ^12,13^, Kelly Cho ^14,15^, Peter WF Wilson ^16,17^, Themistocles L Assimes ^12,13^,

Yan V Sun ^16,18^, Christopher J O’Donnell ^14,15,19^, VA Million Veteran Program,

Marijana Vujkovic ^6,7,*^, Benjamin F Voight ^2,3,4,6,*^

^1^Division of Neonatology, Children’s Hospital of Philadelphia, Philadelphia, PA, USA, 19104

^2^Department of Systems Pharmacology and Translational Therapeutics, Perelman School of Medicine, University of Pennsylvania, Philadelphia, PA, USA, 19104

^3^Department of Genetics, Perelman School of Medicine, University of Pennsylvania, Philadelphia, PA, USA, 19104

^4^Institute of Translational Medicine and Therapeutics, Perelman School of Medicine, University of Pennsylvania, Philadelphia, PA, USA, 19104

^5^Department of Biostatistics, Epidemiology and Informatics, Perelman School of Medicine, University of Pennsylvania, Philadelphia, PA, USA, 19104

^6^Corporal Michael J Crescenz VA Medical Center, Philadelphia, PA, USA, 19104

^7^Department of Medicine, Perelman School of Medicine, University of Pennsylvania, Philadelphia, PA, USA, 19104

^8^Division of Cardiovascular Medicine, Perelman School of Medicine, University of Pennsylvania, Philadelphia, PA, USA, 19104

^9^Department of Surgery, Perelman School of Medicine, University of Pennsylvania, Philadelphia, PA, USA, 19104

﻿^10^VA Informatics and Computing Infrastructure, VA Salt Lake City Health Care System, Salt Lake City, UT, USA, 84148

^11^University of Massachusetts College of Nursing & Health Sciences, Boston, MA, USA, 02125

﻿^12^VA Palo Alto Health Care System, Palo Alto, CA, USA, 94304

^13^Department of Medicine, Stanford University School of Medicine, Stanford, CA, USA, 94305

﻿^14^VA Boston Healthcare System, Boston, MA, USA, 02130

﻿^15^Department of Medicine, Brigham Women’s Hospital, Boston, MA, USA, 02115

﻿^16^Atlanta VA Health Care System, Decatur, GA, USA, 30033

﻿^17^Division of Cardiology, Emory University School of Medicine, Atlanta, GA, USA, 30322

﻿^18^Department of Epidemiology, Emory University Rollins School of Public Health, Atlanta, GA, USA, 30322

^19^Department of Medicine, Harvard Medical School, Boston, MA, USA, 02115

^+,*^: These authors contributed equally to the work

Corresponding author:

Benjamin F Voight

University of Pennsylvania Perelman School of Medicine

3400 Civic Center Blvd

10-126 Smilow Center for Translational Research

Philadelphia, PA 19104

bvoight@pennmedicine.upenn.edu

Phone: 215-746-8083

Fax: 215-573-9135

**Supplemental Material**

**Supplemental Figures**

**
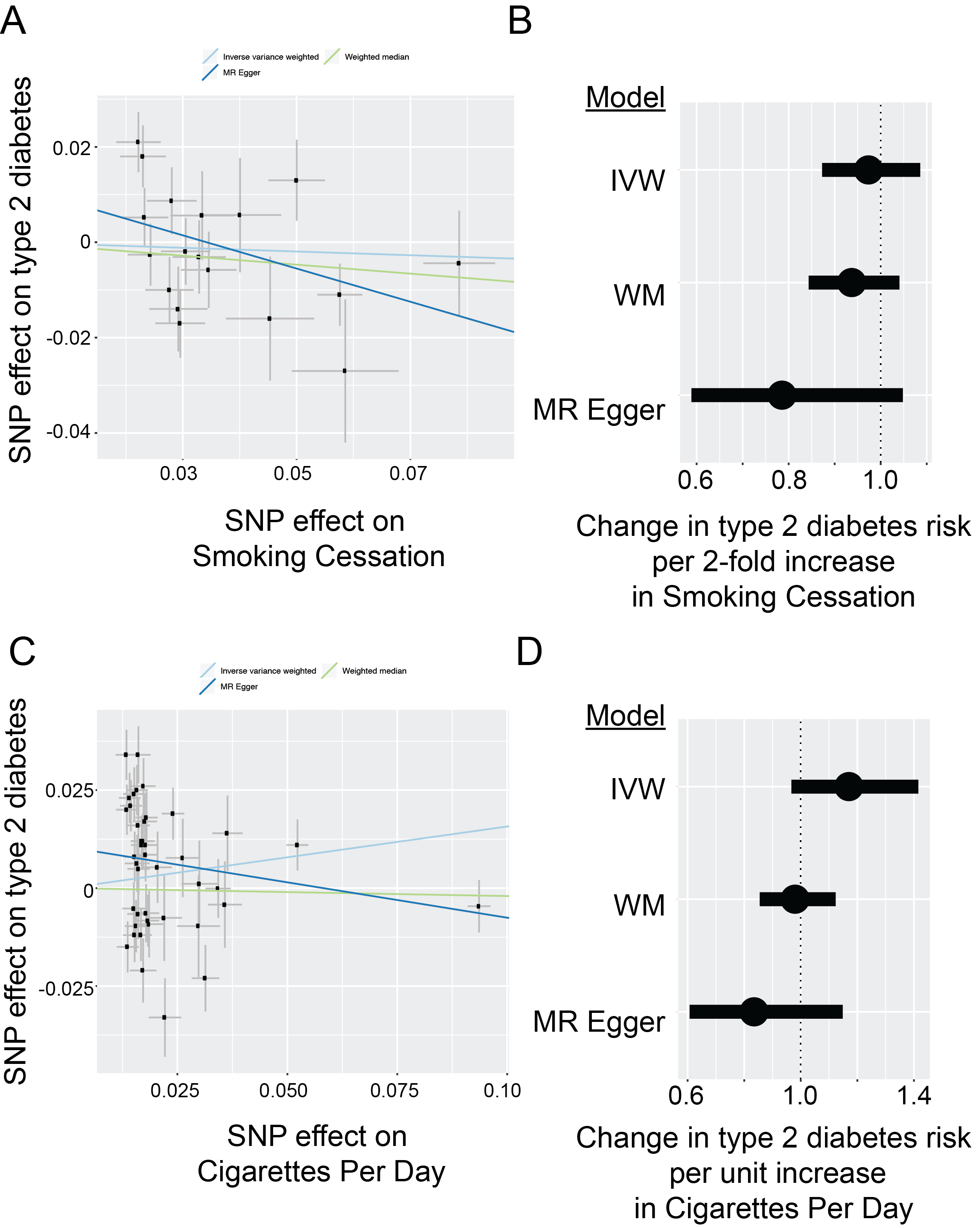
**

**Supplemental Figure 1.**

Two-sample mendelian randomization shows no significant effects of smoking cessation or smoking frequency on type 2 diabetes risk.

(**A**) Using an 18-SNP instrumental variable for smoking cessation, two-sample MR does not define an increased risk of type 2 diabetes. Scatter plot shows effect sizes (mean ± standard error) for smoking cessation and type 2 diabetes for each SNP in the instrumental variable, along with line of best fit for inverse variance weighted, weighted median, and MR-Egger regression models. (**B**) Forest plot showing aggregated effects for smoking cessation on type 2 diabetes, shown as change in type 2 diabetes risk (odds ratio) per 2-fold change in smoking cessation “risk” (mean ± standard error) for inverse variance weighted (IVW), weighted median (WM), and MR-Egger regression models. (**C**) Using a 42-SNP instrumental variable for smoking frequency (number of cigarettes per day), two-sample MR does not define an increased risk of type 2 diabetes. Scatter plot shows effect sizes (mean ± standard error) for smoking frequency and type 2 diabetes for each SNP in the instrumental variable, along with line of best fit for inverse variance weighted, weighted median, and MR-Egger regression models. (**D**) Forest plot showing aggregated instrumental variable effects for smoking frequency on type 2 diabetes, shown as change in type 2 diabetes risk (odds ratio) per unit increase in smoking frequency (mean ± standard error) for inverse variance weighted (IVW), weighted median (WM), and MR-Egger regression models.


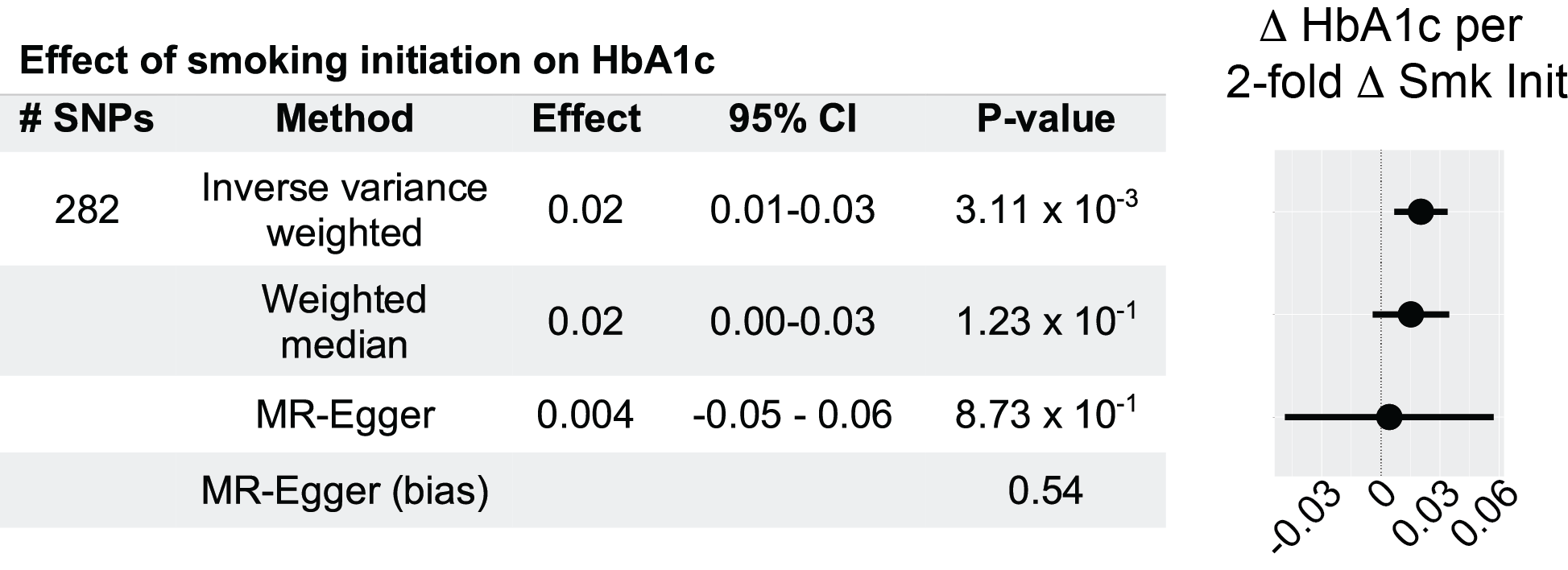


**Supplemental Figure 2**

Increased smoking initiation risk elevates HbA1c by inverse variance weighted method.

Effect estimates, 95% confidence interval, and forest plot represent changes in HbA1c (standard deviation units) associated with 2-fold increase in smoking initiation exposure. MR-Egger intercept does not deviate significantly from zero, validating the effect estimate.


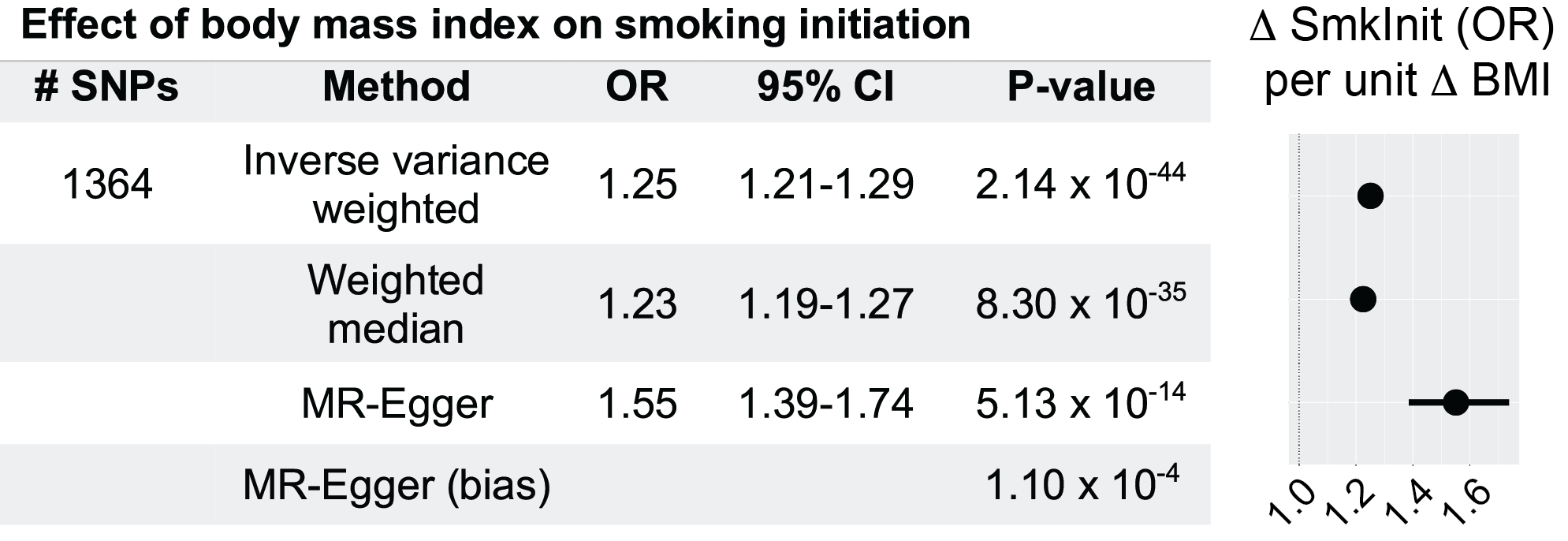


**Supplemental Figure 3**

Genetically determined body mass index (BMI) is associated with increased smoking initiation.

Effect estimates, 95% confidence intervals, and forest plot for smoking initiation ‘risk’ represent changes in odds ratio (OR) for smoking initiation per unit increase in BMI. The MR-Egger intercept deviates significantly from zero, invalidating the effect estimate. The statistically significant effects seen remain valid.


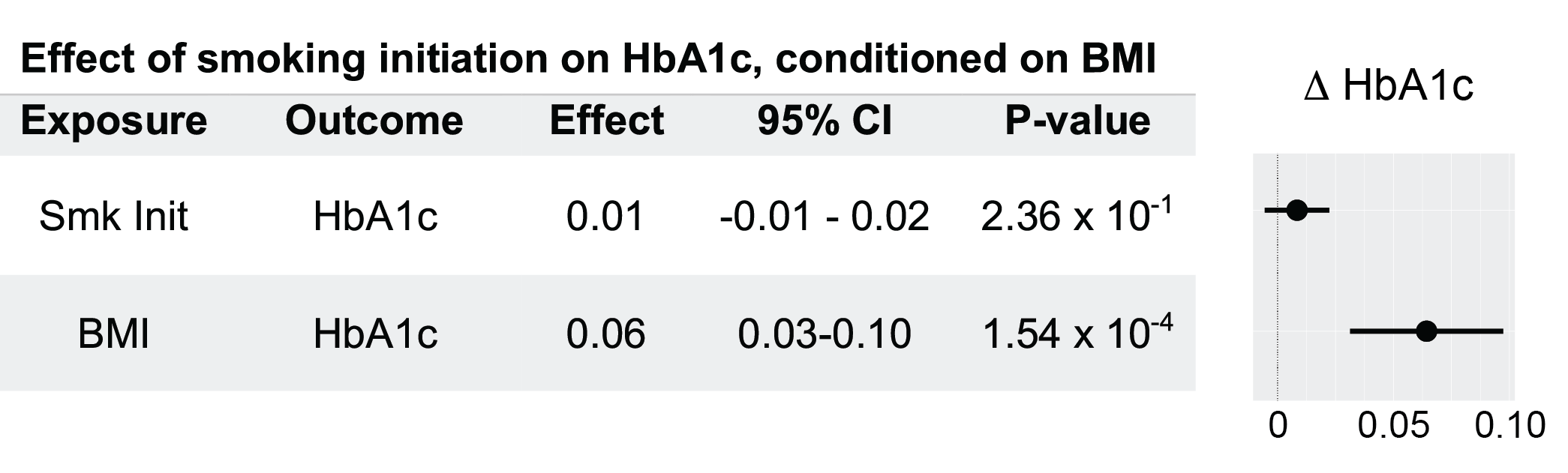


**Supplemental Figure 4**

Body mass index (BMI) mediates the effect of smoking initiation on HbA1c.

Multivariable mendelian randomization (MVMR) results show that genetically determined BMI accounts for the effect of smoking initiation on HbA1c. Effect estimates, 95% confidence intervals, and forest plot represent changes in HbA1c (standard deviation units) per 2-fold increase in genetically determined smoking initiation risk, conditioned on BMI.


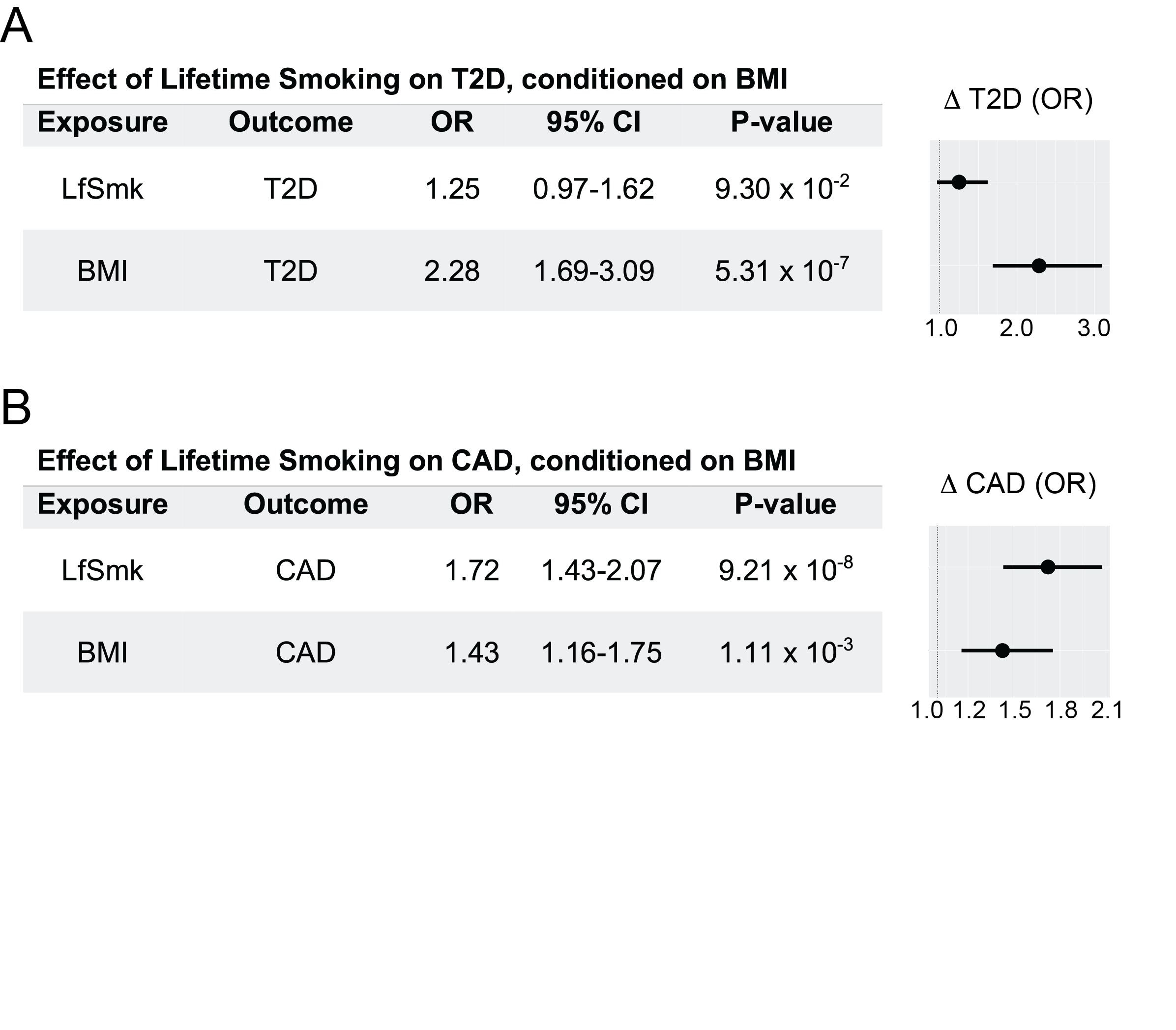


**Supplemental Figure 5**

Body mass index (BMI) mediates the effect of smoking, as defined by Lifetime Smoking score (LfSmk), on increased type 2 diabetes (T2D) risk, but not coronary artery disease (CAD) risk.

Instrumental variables for these experiments comprised 101 SNPs with calculated Lifetime smoking scores (22). (**A**) Multivariable mendelian randomization (MVMR) results show that genetically determined BMI accounts for the effect of lifetime smoking score (LfSmk) on T2D risk. (**B**) Multivariable mendelian randomization (MVMR) results show that BMI and LfSmk have independent effects on CAD risk. Effect and odds ratio (OR) estimates, 95% confidence intervals, and forest plots represent changes per standard deviation unit increase in LfSmk, conditioned on BMI.


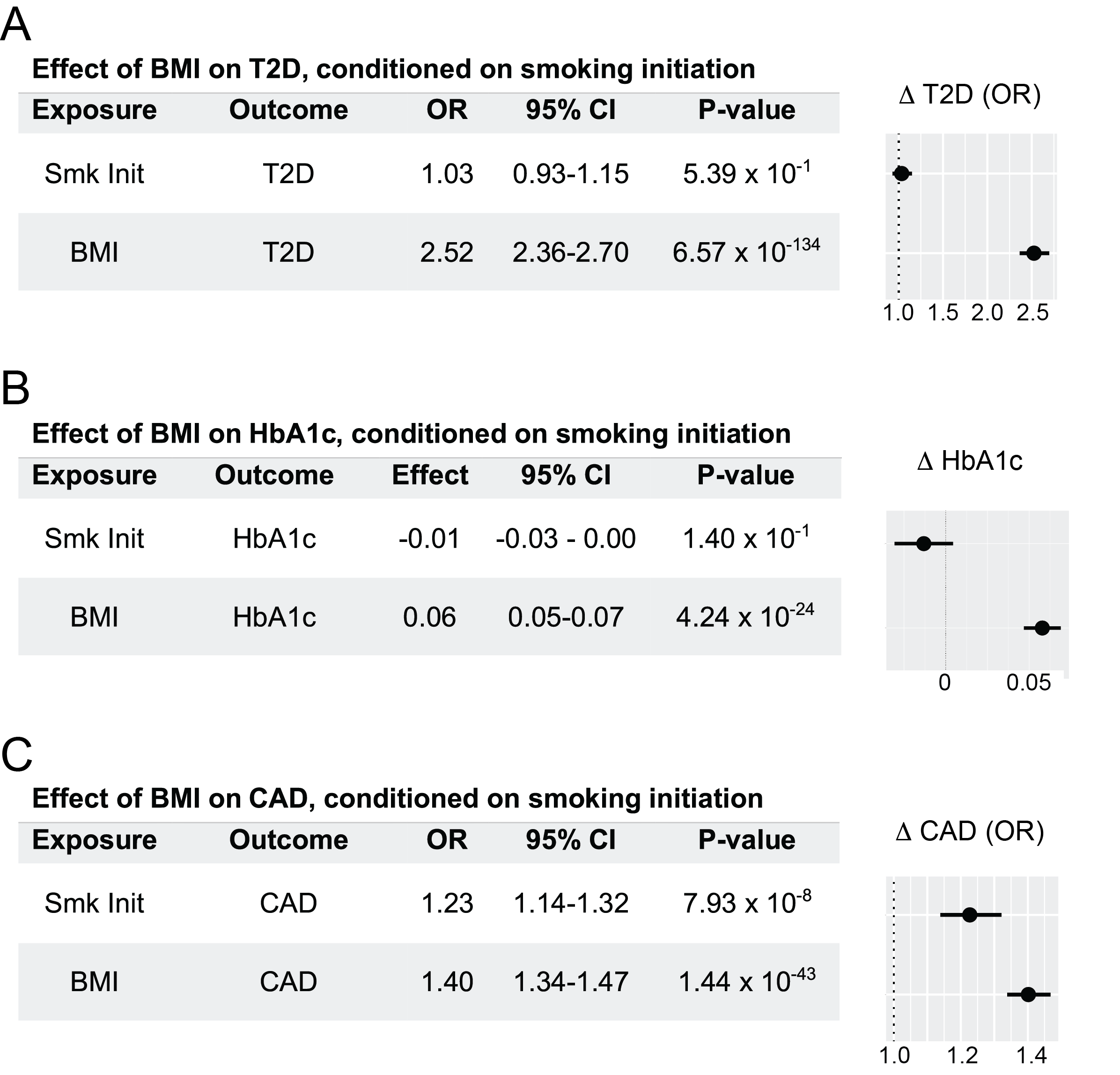


**Supplemental Figure 6**

Body mass index (BMI) mediates the effect of smoking initiation on increased type 2 diabetes (T2D) risk, but not coronary artery disease (CAD) risk.

Instrumental variables for these experiments comprised ~1385 SNPs from BMI GWAS summary statistics (27). (**A**) Multivariable mendelian randomization (MVMR) results show that genetically determined BMI accounts for the effect of smoking initiation on T2D risk. (**B**) Multivariable mendelian randomization (MVMR) results show that genetically determined BMI accounts for the effect of smoking initiation on HbA1c (standard deviation units). (**C**) Multivariable mendelian randomization (MVMR) results show that BMI and smoking initiation have independent effects on CAD risk. Effect and odds ratio (OR) estimates, 95% confidence intervals, and forest plots represent changes per unit increase in genetically determined BMI, conditioned on smoking initiation risk.

**Supplemental Tables Legends**

**Supplemental Table 1.**

Instrument strength F-statistics and related values from GWAS necessary to perform calculations for Two-sample Mendelian randomization experiments (http://cnsgenomics.com/shiny/mRnd (20)). We calculated the proportion of variance explained for the association between the SNPs and exposure variable per Shim *et al* (41). The type I error rate was set to 0.05 in each calculation.

**Supplemental Table 2.**

Smoking cessation and smoking frequency (Cigarettes per day) do not reach statistically significant effects on type 2 diabetes risk based on inverse variance weighted (IVW), weighted median (WM) or MR-Egger regression metrics. Odds ratio (OR) effects and 95% confidence intervals represent change in type 2 diabetes OR associated with 2-fold increased smoking cessation risk, or change in type 2 diabetes OR per 1 standard deviation unit increase in smoking frequency. Significant MR-Egger regression intercept values that deviate significantly from zero (e.g., Cigarettes per day) invalidate effect estimates.

**Supplemental Table 3**.

Two-sample Mendelian randomization analysis results for lifetime smoking score (22) on type 2 diabetes, coronary artery disease, and body mass index. Odds ratio (OR)/Effect and 95% confidence intervals represent changes in disease risk or body mass index associated with a 1 standard deviation unit increase in lifetime smoking score. Significant MR-Egger regression intercept values that deviate significantly from zero (e.g., type 2 diabetes) invalidate effect estimates.

**Supplemental Table 4.**

Genetic correlations between smoking traits (smoking initiation, smoking cessation, smoking frequency, lifetime smoking score) (18,22), body mass index (BMI) (27), type 2 diabetes (19), HbA1c (24), and coronary artery disease (CAD) (38) based on LD Score Regression (23).

**Supplemental Table 5.**

Multivariable mendelian randomization (MVMR) results using (top) an instrumental variable including 1409 linkage-independent SNPs significant for smoking initiation or BMI, or (bottom) 1439 linkage-independent SNPs significant for lifetime smoking score or BMI, show that genetically determined BMI accounts for the effect of smoking initiation on type 2 diabetes risk. Effect and odds ratio (OR) estimates, as well as 95% confidence intervals, represent changes per 2-fold increased smoking initiation risk or a 1 standard deviation increase in lifetime smoking score.

**Supplemental Table 6.**

Two-sample Mendelian randomization analysis results for smoking initiation (18) or lifetime smoking score (22) on type 2 diabetes (31), BMI (30), or CAD (32) in non-overlapping samples. Odds ratio (OR)/Effect and 95% confidence intervals represent changes in disease risk (OR) or BMI (standard deviation units) associated with a 2-fold increase in smoking initiation risk or 1 standard deviation unit increase in lifetime smoking score. The significant MR-Egger regression intercept value that deviates significantly from zero (lifetime smoking score on type 2 diabetes) invalidates that effect estimate. Results are also presented for reciprocal experiments, with smoking behaviors as outcomes. For these results, odds ratio (OR)/Effect estimates and 95% confidence intervals represent changes in smoking initiation odds or lifetime smoking score associated with a 2-fold increase in type 2 diabetes or CAD risk, or 1 standard deviation unit increase in BMI.

**Supplemental Table 7.**

Multivariable mendelian randomization (MVMR) results using an instrumental variable including (top) 207 SNPs significant for smoking initiation or BMI, or (bottom) 254 SNPs significant for lifetime smoking score or BMI, in non-overlapping data sets show that genetically determined BMI accounts for the effect of smoking behaviors on type 2 diabetes risk. Effect and odds ratio (OR) estimates, as well as 95% confidence intervals, represent changes per 2-fold increase in smoking initiation risk or 1 standard deviation increase in lifetime smoking score, conditioned on BMI.

**Supplemental Table 8.**

Multivariable mendelian randomization (MVMR) results using an instrumental variable including (top) 1410 SNPs significant for smoking initiation or CAD, or (bottom) 1441 SNPs significant for lifetime smoking score or CAD, show that genetically determined BMI does not account for the effect of smoking initiation on CAD risk. Effect and odds ratio (OR) estimates, as well as 95% confidence intervals, represent changes per 2-fold increase in smoking initiation risk or 1 standard deviation increase in lifetime smoking score, conditioned on BMI.

**Supplemental Table 9.**

Multivariable mendelian randomization (MVMR) results using an instrumental variable including (top) 207 SNPs significant for smoking initiation or BMI, or (bottom) 254 SNPs significant for lifetime smoking score or BMI, in non-overlapping data sets show that BMI and smoking behaviors have independent effects on CAD risk. Effect and odds ratio (OR) estimates, as well as 95% confidence intervals, represent changes per 2-fold increase in smoking initiation risk or 1 standard deviation increase in lifetime smoking score, conditioned on BMI.

**Supplemental Table 10.**

Mediation analyses depicting total and direct effects of lifetime smoking score on type 2 diabetes or coronary artery disease (CAD) (17). Results of mediation analysis for smoking initiation or lifetime smoking score on outcomes using non-overlapping data are also shown. Values represent increased odds (OR) of disease (OR) per 2-fold increase in smoking initiation risk or 1 standard deviation increase in lifetime smoking score.

**Supplemental Table 11**.

MR-Steiger results assessing directionality between lifetime smoking score or smoking initiation and BMI. Model sensitivity ratios and corresponding p-values are shown, as are estimates for disease measurement precision that were used in these calculations.

**Supplemental Table 12**.

Individual level Mendelian randomization first stage analysis results showing the effects of smoking initiation- or lifetime smoking score-related genetic risk score on BMI, type 2 diabetes, or CAD. Separate analyses based weighted BMI effects from Yengo *et al* or Locke *et al* are shown. Values represent change in disease risk (odds ratio, OR) or BMI (in units of kg/m^2^) per 2-fold increase in smoking initiation risk or 1 standard deviation increase in lifetime smoking score. The lifetime smoking instrument Nagelkerke’s r^2^ was 0.002, corresponding to an F-statistic = 215.

**Supplemental Table 13**.

Individual level Mendelian randomization second stage analysis results showing the effects of smoking initiation- or lifetime smoking score-related genetic risk score on type 2 diabetes, after adjusting for BMI. Separate analyses based weighted BMI effects from Yengo *et al* or Locke *et al* are shown. Values represent increased type 2 diabetes risk (odds ratio, OR) per 2-fold increase in smoking initiation risk or 1 standard deviation increase in lifetime smoking score.

**Supplemental Table 14**.

Individual level Mendelian randomization second stage analysis results showing the effects of smoking initiation- or lifetime smoking score-related genetic risk score on CAD, after adjusting for BMI. Separate analyses based weighted BMI effects from Yengo *et al* or Locke *et al* are shown. Values represent increased heart disease risk (odds ratio, OR) per 2-fold increase in smoking initiation or 1 standard deviation increase in lifetime smoking score.

**Supplemental Table 15.**

Instrumental variable data for MR experiments estimating effects of smoking initiation on type 2 diabetes (Mahajan *et al*). The rsid (hg19), chromosome, position, effect allele, non-effect allele, effect sizes and standard errors are shown for each SNP.

**Supplemental Table 16.**

Instrumental variable data for MR experiments estimating effects of smoking cessation on type 2 diabetes (Mahajan *et al*). The rsid (hg19), chromosome, position, effect allele, non-effect allele, effect sizes and standard errors are shown for each SNP.

**Supplemental Table 17.**

Instrumental variable data for MR experiments estimating effects of smoking frequency (cigarettes per day) on type 2 diabetes (Mahajan *et al*). The rsid (hg19), chromosome, position, effect allele, non-effect allele, effect sizes and standard errors are shown for each SNP.

**Supplemental Table 18.**

Instrumental variable data for MR experiments estimating effects of smoking initiation on HbA1c. The rsid (hg19), chromosome, position, effect allele, non-effect allele, effect sizes and standard errors are shown for each SNP.

**Supplemental Table 19.**

Instrumental variable data for MR experiments estimating effects of smoking initiation on coronary artery disease (van der Harst & Verweij). The rsid (hg19), chromosome, position, effect allele, non-effect allele, effect sizes and standard errors are shown for each SNP.

**Supplemental Table 20.**

Instrumental variable data for MR experiments estimating effects of smoking initiation on BMI (Yengo *et al*). The rsid (hg19), chromosome, position, effect allele, non-effect allele, effect sizes and standard errors are shown for each SNP.

**Supplemental Table 21.**

Instrumental variable data for MR experiments estimating effects of BMI (Yengo *et al*) on smoking initiation. The rsid (hg19), chromosome, position, effect allele, non-effect allele, effect sizes and standard errors are shown for each SNP.

**Supplemental Table 22.**

Instrumental variable data for MR experiments estimating effects of lifetime smoking score on type 2 diabetes (Mahajan *et al*). The rsid (hg19), chromosome, position, effect allele, non-effect allele, effect sizes and standard errors are shown for each SNP.

**Supplemental Table 23.**

Instrumental variable data for MR experiments estimating effects of lifetime smoking score on BMI (Yengo *et al*). The rsid (hg19), chromosome, position, effect allele, non-effect allele, effect sizes and standard errors are shown for each SNP.

**Supplemental Table 24.**

Instrumental variable data for MR experiments estimating effects of lifetime smoking score on coronary artery disease (CAD, van der Harst & Verweij). The rsid (hg19), chromosome, position, effect allele, non-effect allele, effect sizes and standard errors are shown for each SNP.

**Supplemental Table 25.**

Instrumental variable data for MR experiments estimating effects of lifetime smoking score on type 2 diabetes (Mahajan *et al*), conditioned on BMI (Yengo *et al*). The rsid (hg19), chromosome, position, effect allele, non-effect allele, effect sizes and standard errors are shown for each SNP.

**Supplemental Table 26.**

Instrumental variable data for MR experiments estimating effects of lifetime smoking on CAD (van der Harst & Verweij), conditioned on BMI (Yengo *et al*). The rsid (hg19), chromosome, position, effect allele, non-effect allele, effect sizes and standard errors are shown for each SNP.

**Supplemental Table 27.**

Instrumental variable data for MR experiments estimating effects of smoking initiation on HbA1c, conditioned on BMI (Yengo *et al*). The rsid (hg19), chromosome, position, effect allele, non-effect allele, effect sizes and standard errors are shown for each SNP.

**Supplemental Table 28.**

Instrumental variable data for MR experiments estimating effects of smoking initiation on type 2 diabetes (Mahajan *et al*), conditioned on BMI (Yengo *et al*). The rsid (hg19), chromosome, position, effect allele, non-effect allele, effect sizes and standard errors are shown for each SNP.

**Supplemental Table 29.**

Instrumental variable data for MR experiments estimating effects of smoking initiation on CAD (van der Harst & Verweij), conditioned on BMI (Yengo *et al*). The rsid (hg19), chromosome, position, effect allele, non-effect allele, effect sizes and standard errors are shown for each SNP.

**Supplemental Table 30.**

Instrumental variable data for MR experiments estimating effects of BMI (Yengo *et al*) on type 2 diabetes (Mahajan *et al*), conditioned on smoking initiation. The rsid (hg19), chromosome, position, effect allele, non-effect allele, effect sizes and standard errors are shown for each SNP.

**Supplemental Table 31.**

Instrumental variable data for MR experiments estimating effects of BMI (Yengo *et al*) on HbA1c, conditioned on smoking initiation. The rsid (hg19), chromosome, position, effect allele, non-effect allele, effect sizes and standard errors are shown for each SNP.

**Supplemental Table 32.**

Instrumental variable data for MR experiments estimating effects of BMI (Yengo *et al*) on CAD (van der Harst & Verweij), conditioned on smoking initiation. The rsid (hg19), chromosome, position, effect allele, non-effect allele, effect sizes and standard errors are shown for each SNP.

**Supplemental Table 33.**

Instrumental variable data for MR experiments estimating effects of smoking initiation on type 2 diabetes (Scott *et al*, non-overlapping samples). The rsid (hg19), chromosome, position, effect allele, non-effect allele, effect sizes and standard errors are shown for each SNP.

**Supplemental Table 34.**

Instrumental variable data for MR experiments estimating effects of smoking initiation on CAD (Nikpay *et al*, non-overlapping samples). The rsid (hg19), chromosome, position, effect allele, non-effect allele, effect sizes and standard errors are shown for each SNP.

**Supplemental Table 35.**

Instrumental variable data for MR experiments estimating effects of smoking initiation on BMI (Locke *et al*, non-overlapping samples). The rsid (hg19), chromosome, position, effect allele, non-effect allele, effect sizes and standard errors are shown for each SNP.

**Supplemental Table 36.**

Instrumental variable data for MR experiments estimating effects of type 2 diabetes (Scott *et al*) on smoking initiation (non-overlapping samples). The rsid (hg19), chromosome, position, effect allele, non-effect allele, effect sizes and standard errors are shown for each SNP.

**Supplemental Table 37.**

Instrumental variable data for MR experiments estimating effects of CAD (Nikpay *et al*) on smoking initiation (non-overlapping samples). The rsid (hg19), chromosome, position, effect allele, non-effect allele, effect sizes and standard errors are shown for each SNP.

**Supplemental Table 38.**

Instrumental variable data for MR experiments estimating effects of BMI (Locke *et al*) on smoking initiation (non-overlapping samples). The rsid (hg19), chromosome, position, effect allele, non-effect allele, effect sizes and standard errors are shown for each SNP.

**Supplemental Table 39.**

Instrumental variable data for MR experiments estimating effects of lifetime smoking score on type 2 diabetes (Scott *et al*, non-overlapping samples). The rsid (hg19), chromosome, position, effect allele, non-effect allele, effect sizes and standard errors are shown for each SNP.

**Supplemental Table 40.**

Instrumental variable data for MR experiments estimating effects of lifetime smoking score on CAD (Nikpay *et al*, non-overlapping samples). The rsid (hg19), chromosome, position, effect allele, non-effect allele, effect sizes and standard errors are shown for each SNP.

**Supplemental Table 41.**

Instrumental variable data for MR experiments estimating effects of lifetime smoking score on BMI (Locke *et al*, non-overlapping samples). The rsid (hg19), chromosome, position, effect allele, non-effect allele, effect sizes and standard errors are shown for each SNP.

**Supplemental Table 42.**

Instrumental variable data for MR experiments estimating effects of type 2 diabetes (Scott *et al*) on lifetime smoking score (non-overlapping samples). The rsid (hg19), chromosome, position, effect allele, non-effect allele, effect sizes and standard errors are shown for each SNP.

**Supplemental Table 43.**

Instrumental variable data for MR experiments estimating effects of CAD (Nikpay *et al*) on lifetime smoking score (non-overlapping samples). The rsid (hg19), chromosome, position, effect allele, non-effect allele, effect sizes and standard errors are shown for each SNP.

**Supplemental Table 44.**

Instrumental variable data for MR experiments estimating effects of BMI (Locke *et al*) on lifetime smoking score (non-overlapping samples). The rsid (hg19), chromosome, position, effect allele, non-effect allele, effect sizes and standard errors are shown for each SNP.

**Supplemental Table 45.**

Instrumental variable data for MR experiments estimating effects of smoking initiation on type 2 diabetes (Scott *et al*), conditioned on BMI (Locke *et al*, non-overlapping samples). The rsid (hg19), chromosome, position, effect allele, non-effect allele, effect sizes and standard errors are shown for each SNP.

**Supplemental Table 46.**

Instrumental variable data for MR experiments estimating effects of smoking initiation on CAD (Nikpay *et al*), conditioned on BMI (Locke *et al*, non-overlapping samples). The rsid (hg19), chromosome, position, effect allele, non-effect allele, effect sizes and standard errors are shown for each SNP.

**Supplemental Table 47.**

Instrumental variable data for MR experiments estimating effects of lifetime smoking score on type 2 diabetes (Scott *et al*), conditioned on BMI (Locke *et al*, non-overlapping samples). The rsid (hg19), chromosome, position, effect allele, non-effect allele, effect sizes and standard errors are shown for each SNP.

**Supplemental Table 48.**

Instrumental variable data for MR experiments estimating effects of lifetime smoking score on CAD (Nikpay *et al*), conditioned on BMI (Locke *et al*, non-overlapping samples). The rsid (hg19), chromosome, position, effect allele, non-effect allele, effect sizes and standard errors are shown for each SNP.
